# Cardiorespiratory tolerance of continuous dexmedetomidine infusion in preterm and term newborn infants: a retrospective cohort study

**DOI:** 10.1007/s00431-026-07000-7

**Published:** 2026-05-02

**Authors:** Mathilde Blouin, Sixtine Gilliot, Anne Wojtanowski, Julien De Jonckheere, Laurent Storme, Bertrand Decaudin, Pascal Odou, Kevin Le Duc, Mohamed Riadh Boukhris

**Affiliations:** 1 Department of Neonatology, CHU Lille, Jeanne de Flandre Hospital, Lille, F‑59000 France; 2 Pharmacy Institute, CHU Lille, Lille, F‑59000 France; 3 Univ. Lille, CHU Lille, ULR 7365 - GRITA - Groupe de Recherche sur les formes Injectables et les Technologies Associées, Lille, F-59000 France; 4 CHU Lille, CIC-IT 1403, Lille, F‑59000 France; 5 Univ. Lille, CHU Lille, ULR 2694- METRICS : Évaluation des technologies de santé et des pratiques médicales, Lille, F‑59000 France

**Keywords:** Dexmedetomidine, Neonate, Preterm infant, Sedation, Bradycardia, Intensive care units

## Abstract

Dexmedetomidine (DEX) is increasingly used for neonatal sedation, but safety data remain limited. We conducted a single-center retrospective study including neonates receiving continuous DEX infusion. Cardiorespiratory events were extracted from bedside monitoring during the 8 h before and the 24 h after initiation. Hemodynamic and clinical parameters were analyzed, and autonomic activity was assessed using Newborn Infant Parasympathetic Evaluation (NIPE) monitoring in a subgroup. Thirty-seven infants (18 preterm, 19 term) were included; 86% received concomitant morphine. Bradycardia episodes increased after DEX initiation, particularly in preterm infants (*p* < 0.05). In contrast, hypotension, lactate levels remained unchanged, while urine output varied over time without a clinically meaningful reduction. Hypoxemic events decreased, while oxygen requirements remained stable. In the NIPE subgroup, heart rate decreased, with a trend toward increased NIPE values. DEX was associated with increased bradycardia without clear evidence of impaired hemodynamic or respiratory tolerance. These findings suggest an overall reassuring short-term safety profile and suggest a physiologically mediated sedative effect.

**Table:** 

**What is Known:** • *Dexmedetomidine is increasingly used for sedation in preterm and term neonates, but cardiorespiratory safety data remain limited.* • *Bradycardia is the most frequently reported adverse effect.* **What is New:** • *Continuous monitor-derived data show increased bradycardia after dexmedetomidine initiation, without hypotension or impaired perfusion, while hypoxemic events decreased.* • *Autonomic monitoring (NIPE) suggests a trend toward increased parasympathetic activity, which may reflect modulation of autonomic balance under dexmedetomidine.*

**Supplementary information:**

The online version contains supplementary material available at 10.1007/s00431-026-07000-7.

## Introduction

Pain management in the neonatal intensive care unit (NICU) remains a major clinical challenge [1]. Experimental and clinical studies have shown that repeated painful stimuli during early life may adversely affect brain development, particularly in preterm infants [2]. Although non-pharmacological interventions and acetaminophen are frequently used, they are often insufficient in critically ill neonates requiring invasive ventilation, surgery, or repeated procedures [2].

Opioids remain the most commonly administered sedative and analgesic agents in this population. However, their use is associated with important adverse effects, including respiratory depression, gastrointestinal dysmotility, and withdrawal syndrome, which may contribute to additional morbidity, especially in preterm infants [3]. Preclinical data have also raised concerns regarding potential neurotoxicity and altered brain development following early opioid exposure [4].

Dexmedetomidine (DEX), a highly selective α₂-adrenergic receptor agonist, provides analgesia, sedation, and anxiolysis through central sympatholytic effects [5]. Unlike opioids and benzodiazepines, DEX preserves respiratory drive and gastrointestinal motility. In pediatric and adult intensive care, it has been associated with reduced opioid requirements and shorter durations of mechanical ventilation [6]. Neuroprotective effects have also been suggested in animal models [7]. Its use in neonatal practice has increased in recent years despite limited safety data. Nevertheless, α₂-mediated sympatholysis may induce bradycardia and hypotension [8], and available neonatal studies remain limited, particularly in preterm infants and regarding dosing strategies [5, 6].

The objective of this study was to evaluate the cardiorespiratory tolerance of the initiation of a continuous infusion treatment with DEX in neonates admitted to the NICU.

## Materials and methods

We conducted a retrospective cohort study in a level III NICU at the University Hospital of Lille (France) between December 2021 and May 2022. According to national regulations, this study based on routinely collected data did not require formal ethical approval, and all data protection requirements were fulfilled.

DEX was initiated when first-line analgesia, consisting of acetaminophen and opioids, failed to provide adequate comfort based on behavioral scales (EDIN score > 5 or COMFORT Behavior score > 18). According to the unit protocol, DEX was administered as a continuous intravenous infusion starting at 0.4 µg/kg/h and increased by 0.2 µg/kg/h every 4 h if needed, up to a maximum dose of 1.4 µg/kg/h. In cases of bradycardia (heart rate < 80 bpm in term infants or < 90 bpm in preterm infants), the infusion rate was reduced by 0.2 µg/kg/h.

All consecutive neonates receiving continuous DEX infusion for at least 8 h were eligible. Each treatment course was considered an independent inclusion event. Infants treated with benzodiazepines or undergoing therapeutic hypothermia were excluded because of their potential effects on heart rate and hemodynamic parameters. Concomitant opioid therapy was allowed, reflecting routine practice.

Baseline characteristics included sex, gestational age, birth weight, respiratory support, FiO₂, pre-ductal SpO_2_, urine output, plasma lactate concentration, and concomitant medications (opioids and vasopressors). Lactate levels were routinely measured every 8 h. The pre-ductal SpO2/FiO2 (S/F) ratio was calculated for each study period. DEX-related variables included starting dose, duration of therapy, and maximum dose during the first 24 h. Opioid doses were converted to morphine equivalents.

Infants were continuously monitored using Philips IntelliVue systems for heart rate, pulse oximetry, and non-invasive blood pressure. Alarm data were automatically extracted. Bradycardia was defined as heart rate < 80 bpm in term infants and < 90 bpm in preterm infants, with severe bradycardia defined as an additional decrease of > 10 bpm. Hypotension was defined as mean blood pressure below gestational age thresholds, and hypoxemia as pre-ductal SpO₂ < 88%.

Episodes occurring within one minute (bradycardia or hypoxemia) or 15 min (hypotension) were merged into a single event. Events were quantified during the 8 h preceding DEX initiation (H − 8/H0) and during three consecutive 8-h periods thereafter (H0/H8, H8/H16, H16/H24).

When available, autonomic activity was assessed using the Newborn Infant Parasympathetic Evaluation (NIPE) monitor, which evaluates parasympathetic tone based on heart rate variability [9].

Continuous variables are reported as median (interquartile range). Non-parametric tests were used. Event frequencies were compared using Friedman tests followed by Wilcoxon signed-rank tests with Bonferroni correction (*p* < 0.0125). Comparisons between term and preterm infants were performed using Mann–Whitney tests. Correlations between cumulative opioid dose and adverse events were assessed using Pearson correlation. Subgroup analyses were conducted according to gestational age, with additional exploratory analyses in the NIPE subgroup. Statistical analyses were performed using SPSS version 20 (IBM Corp.).

## Results

### Population characteristics

During the study period, 282 infants were admitted to the NICU, of whom 67 received DEX. Thirty were excluded (11 midazolam, 7 therapeutic hypothermia, 12 missing data), leaving 37 infants (18 preterm and 19 term) for analysis.

Median gestational age at birth was 37 weeks (IQR 30–39), and median birth weight 2600 g (IQR 1650–3120). DEX was initiated at a median postnatal age of 2 days (IQR 1–20) and continued for 3 days (IQR 2–5).

Fifty-one percent of infants were post-operative (mainly esophageal atresia and intestinal obstruction), 89% required invasive ventilation, and 14% received vasoactive support at initiation. Most infants (86%) received morphine. The interval between opioid initiation and DEX initiation was longer in preterm infants than in term infants (median 16.5 vs 3 h, *p* = 0.016).

Detailed baseline characteristics are provided in Supplementary online materials.

### Hemodynamic outcomes

**Fig. 1:**
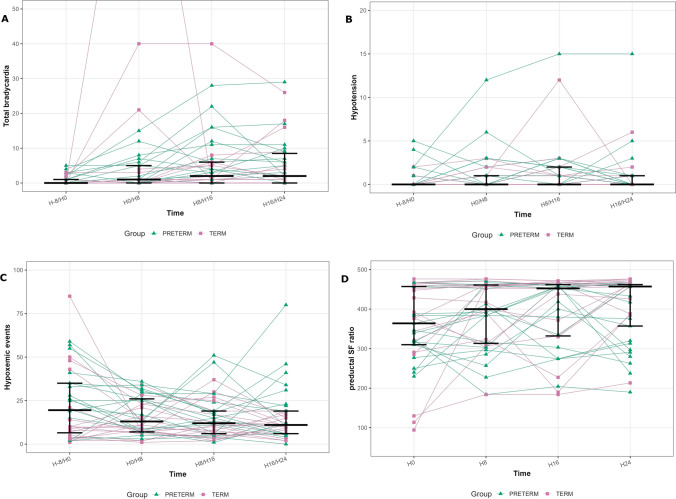
Cardiorespiratory events before and after dexmedetomidine initiation. Longitudinal evolution of **A** total bradycardia episodes, **B** hypotension, **C** hypoxemic events, and **D** pre-ductal SpO_2_/FiO_2_ (S/F) ratio during the 8 h before dexmedetomidine initiation and the first 24 h thereafter. Individual trajectories are shown across four consecutive 8-h intervals: H-8/0 H0/H8, H8/H16 and H16/H24. Green triangles indicate preterm infants and pink squares indicate term infants. Black horizontal bars represent median values with interquartile ranges. Bradycardia was defined as heart rate < 90 bpm in preterm infants and < 80 bpm in term infants. Hypoxemia was defined as pre-ductal SpO_2_ < 88%, and hypotension as mean arterial pressure below gestational age thresholds

Bradycardia episodes increased after DEX initiation, from 0 (0–1) to 1 (0–5.75), 2 (0–7.75), and 2 (0–8.5) across time periods (*p* < 0.001) (Fig. 1). Severe bradycardias showed a similar pattern, more marked in preterm infants.

Hypotension was infrequent and unchanged (*p* = 0.175), with no treatment discontinuation. Plasma lactate concentrations remained stable (*p* = 0.148). Urine output showed a statistically significant variation over time (*p* = 0.023), although without clinically meaningful reduction. No correlation was observed between opioid exposure and adverse events.

Five infants (13.5%) received vasoactive support (dopamine *n* = 3, dobutamine *n* = 1, norepinephrine *n* = 1, milrinone *n* = 2). Exposure was heterogeneous and mostly transient, with early discontinuation in several cases, and dose escalation in only one infant.

### Respiratory outcomes

Hypoxemic episodes decreased after DEX initiation, from 19 (5–35) to 11 (7–26) during H0–H8 and remained lower thereafter (*p* = 0.016). FiO₂ remained stable (*p* = 0.11), with unchanged SpO2. The pre-ductal S/F ratio remained stable over time (Fig. 1).

Among ventilated infants, 30% were extubated within 24 h.

### Parasympathetic evaluation

**Fig. 2:**
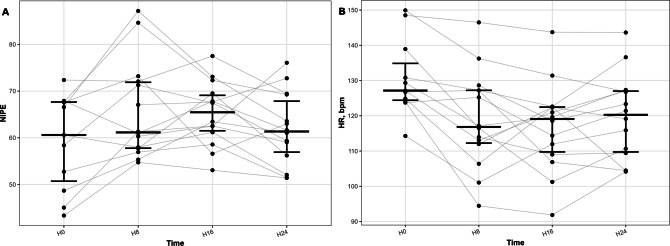
Evolution of heart rate and autonomic activity during dexmedetomidine initiation (NIPE subgroup). Longitudinal evolution of **A** heart rate and **B** Newborn Infant Parasympathetic Evaluation (NIPE) values during the 8 h before dexmedetomidine initiation (H − 8/H0) and the 24 h thereafter. Individual trajectories are shown across four consecutive 8-h intervals: H-8/0 H0/H8, H8/H16 and H16/H24. Black horizontal bars represent median values with interquartile ranges

NIPE monitoring was available in 11 infants (30%). Heart rate decreased significantly (127 to 117–121 bpm during follow-up; *p* ≤ 0.033), while NIPE values tended to increase at H8 and H16, although differences were not statistically significant (Fig. 2).

## Discussion

In this retrospective cohort of preterm and term neonates, initiation of continuous DEX infusion was associated with an increased incidence of bradycardia, without clinically significant hypotension or evidence of impaired perfusion. Hypoxemic episodes decreased following DEX initiation. Together, these findings support acceptable short-term cardiorespiratory tolerance of DEX in critically ill neonates.

Bradycardia was the main hemodynamic effect and is consistent with the known pharmacological action of α₂-adrenergic agonists, which reduce sympathetic tone and enhance parasympathetic activity [8]. Importantly, it was not associated with hypotension or indicators of hypoperfusion, suggesting a pharmacodynamic rather than pathological mechanism. These findings align with previous studies describing bradycardia as the most frequent but generally well-tolerated adverse effect [10–12].

A novel aspect of this study is the integration of autonomic monitoring using NIPE. Following DEX initiation, heart rate decreased while NIPE values tended to increase, indicating enhanced parasympathetic activity. As NIPE reflects heart rate variability and has been proposed as a marker of comfort [13], these findings provide physiological support for effective sedation under DEX. Importantly, this autonomic profile reinforces the interpretation of bradycardia as a pharmacodynamic effect rather than a sign of cardiovascular compromise. This combined analysis of cardiorespiratory and autonomic data offers a more integrated understanding of DEX effects in neonates, beyond conventional clinical parameters. To our knowledge, such continuous autonomic assessment has rarely been reported in this context.

DEX may offer potential respiratory advantages compared with opioids and benzodiazepines, as it does not suppress respiratory drive. In our study, respiratory tolerance was preserved, with stable oxygen requirements, decreased hypoxemic events, and unchanged S/F ratio. Although causality cannot be established, these findings are consistent with previous reports describing minimal respiratory depression and reduced exposure to other sedatives with DEX [14, 15].

Strengths of this study include the use of continuous monitoring data and a within-patient design, limiting interindividual variability. The integration of NIPE provides additional physiological insight into sedation effects. Limitations include the retrospective, single-center design and small sample size. Concomitant opioid therapy and occasional vasoactive support may have influenced the results, although no systematic increase in vasoactive support was observed. The restricted study period may also limit generalizability.

## Conclusion

Continuous DEX infusion was associated with increased bradycardia but preserved hemodynamic stability and respiratory tolerance in preterm and term neonates. These findings suggest that DEX appears to be well tolerated for sedation in the NICU, although prospective studies are warranted to confirm safety and define optimal dosing strategies.

## Supplementary information

Below is the link to the electronic supplementary material.

ESM 1 (DOCX 296 KB)

## Data Availability

The dataset generated and/or analyzed during the current study are available from the corresponding author on reasonable request.
