# Supplementary material for: Cardiorespiratory tolerance of continuous dexmedetomidine infusion in preterm and term newborn infants: a retrospective cohort study
Source: Eur J Pediatr. 2026 May 2;185(5):337. doi: 10.1007/s00431-026-07000-7 (PMC13135545; doi:10.1007/s00431-026-07000-7)
Supplement: Supplementary file 1 — (DOCX 296 KB) [file 431_2026_7000_MOESM1_ESM.docx]

**Tables**

**Table 1. Baseline characteristics of the study population**

| Characteristic | Overall (n=37) | Preterm (n=18) | Term (n=19) | p-value |
| --- | --- | --- | --- | --- |
| Gestational age at birth, weeks | 37 (30–39) | 30 (27–35) | 39 (38–40) | — |
| Male sex, n (%) | 23 (62) | 12 (67) | 11 (58) | 0.582 |
| Birth weight, g | 2600 (1650–3120) | 1615 (685–2100) | 3120 (2890–3415) | <0.001 |
| Postnatal age at DEX initiation, days | 2 (1–20) | 6 (2–23) | 2 (1–4) | 0.057 |
| Postmenstrual age at DEX initiation, weeks | 38 (35–40) | 35 (31–36) | 40 (39–41) | — |
| Weight at DEX initiation, g | 2700 (2100–3350) | 2085 (1540–2600) | 3290 (3100–3495) | <0.001 |
| Surgical patient, n (%) | 19 (51) | 6 (33) | 13 (68) | 0.048 |
| Invasive ventilation, n (%) | 33 (89) | 17 (94) | 16 (84) | 0.316 |
| Death during hospitalization, n (%) | 3 (8) | 2 (11) | 1 (5) | 0.604 |
| Inotropes/vasopressors, n (%) | 5 (14) | 4 (22) | 1 (5) | 0.162 |
| Morphine, n (%) | 32 (86) | 17 (94) | 15 (79) | 0.340 |
| Sufentanil, n (%) | 5 (14) | 1 (6) | 4 (21) | 0.340 |
| Opioid dose at initiation, µg/kg/h | 20 (20–30) | 25 (20–30) | 20 (20–30) | 0.407 |
| Cumulative opioid dose before DEX, µg/kg | 17 (8–24) | 20 (10–26) | 17 (3–20) | 0.313 |
| Time between opioid and DEX initiation, h | 12 (2.5–28) | 16.5 (9–64) | 3 (1–16) | 0.016 |
| Duration of DEX treatment, days | 3 (2–5) | 2.5 (1–5) | 4 (2–5) | 0.538 |
| Maximum DEX dose during first 24 h, µg/kg/h | 0.4 (0.4–0.6) | 0.4 (0.4–0.4) | 0.4 (0.4–0.7) | 0.169 |

*Data are presented as median (IQR) or n (%).*

**Table 2: indications for analgosedation**

| Indications | N |
| --- | --- |
| Respiratory distress syndrom | **1** |
| Sepsis in premature infants | **7** |
| Esophageal atresia | **7** |
| Intestinal occlusion | **6** |
| Gastroschisis | **1** |
| Intestinal bowel perforation | **2** |
| Meconium aspiration syndrom | **2** |
| Respiratory distress syndrom | **1** |
| Pneumothorax | **1** |
| Chylothorax | **2** |
| Perinatal asphyxia | **2** |
| Pierre Robin sequence | **1** |
| NEC | **1** |
| Cardiomyopathy | **1** |
| Post laser retinopathy | **1** |

**Table 3.** **Cardiorespiratory adverse events before and after dexmedetomidine initiation**

| Event (episodes/8 h) | Time | Overall (n=37) | Preterm (n=18) | Term (n=19) | p-value |
| --- | --- | --- | --- | --- | --- |
| Total bradycardia | H−8/H0 | 0 (0–1) | 0 (0–2.25) | 0 (0–0.75) | 0.188 |
|  | H0/H8 | 1 (0–5.75) | 1 (0–6.25) | 0 (0–3) | 0.316 |
|  | H8/H16 | 2 (0–7.75) | 4 (1–13) | 1 (0–5) | 0.030 |
|  | H16/H24 | 2 (0–8.5) | 5 (1–9.5) | 1 (0–6) | 0.203 |
| Severe bradycardia | H−8/H0 | 0 (0–1) | 0 (0–1) | 0 (0–0) | 0.326 |
|  | H0/H8 | 0 (0–2.5) | 0.5 (0–4.25) | 0 (0–0) | 0.023 |
|  | H8/H16 | 0.5 (0–4.75) | 3.5 (0–9.25) | 0 (0–1) | 0.002 |
|  | H16/H24 | 1.5 (0–3) | 2 (0.5–3.5) | 1 (0–2.25) | 0.103 |
| Hypotension | H−8/H0 | 0 (0–0) | 0 (0–1.25) | 0 (0–0) | 0.231 |
|  | H0/H8 | 0 (0–1) | 0 (0–2.25) | 0 (0–1) | 0.199 |
|  | H8/H16 | 0 (0–1.75) | 1 (0–2.25) | 0 (0–1) | 0.125 |
|  | H16/H24 | 0 (0–1) | 0 (0–1) | 0 (0–0.25) | 0.384 |
| Hypoxemic events | H−8/H0 | 19 (5–35) | 25.5 (18–36.5) | 9.5 (3.75–26.5) | 0.051 |
|  | H0/H8 | 11 (7–26) | 21 (7.75–30.25) | 9 (6–21) | 0.053 |
|  | H8/H16 | 12 (6–24) | 15 (5.75–25.25) | 10 (4–19) | 0.236 |
|  | H16/H24 | 11 (6–19) | 12 (6–32.5) | 11 (8–15) | 0.302 |

*Data are presented as median (IQR).*

**Table 4. Clinical parameters before and after dexmedetomidine initiation**

| Parameter | Time | Overall (n=37) | Preterm (n=18) | Term (n=19) | p-value |
| --- | --- | --- | --- | --- | --- |
| Plasma lactate, mmol/L | H0 | 1.67 (0.84–2.35) | 1.32 (0.84–2.25) | 1.74 (1.34–2.42) | 0.242 |
|  | H8 | 1.33 (0.93–2.38) | 1.33 (1.00–2.00) | 1.20 (0.88–2.23) | 0.678 |
|  | H16 | 1.27 (0.87–2.65) | 1.27 (0.92–4.32) | 1.33 (0.92–1.80) | 0.612 |
|  | H24 | 1.15 (0.62–1.93) | 1.02 (0.74–2.59) | 1.10 (0.61–1.58) | 0.460 |
| Urine output, mL/kg/h | H0 | 2.50 (1.58–4.40) | 3.85 (2.14–4.77) | 2.05 (1.18–3.50) | 0.050 |
|  | H8 | 2.75 (1.44–4.50) | 4.30 (1.43–4.65) | 2.10 (1.20–3.20) | 0.109 |
|  | H16 | 2.50 (1.67–3.45) | 2.80 (2.07–3.75) | 2.10 (1.25–3.20) | 0.066 |
|  | H24 | 3.25 (1.87–4.18) | 3.25 (2.20–3.97) | 3.40 (1.47–4.22) | 0.987 |
| FiO₂, % | H0 | 25 (21–30) | 29 (25–30) | 21 (21–28) | 0.106 |
|  | H8 | 24.5 (21–30) | 25 (21.5–31.5) | 21 (21–27.5) | 0.043 |
|  | H16 | 21 (21–27.25) | 21 (21–27.25) | 21 (21–25) | 0.630 |
|  | H24 | 21 (21–29.5) | 24 (21–31.5) | 21 (21–22.5) | 0.035 |

*Data are presented as median (IQR).*

**Table 5. Autonomic parameters during dexmedetomidine infusion (NIPE subgroup)**

| Parameter | Time | Overall (n=11) | p-value |
| --- | --- | --- | --- |
| Heart rate, bpm | H0 | 127 (124–139) | — |
|  | H8 | 117 (106–127) | 0.008 |
|  | H16 | 120 (107–123) | 0.003 |
|  | H24 | 121 (111–127) | 0.033 |
| NIPE score | H0 | 61 (49–68) | — |
|  | H8 | 61 (57–73) | 0.033 |
|  | H16 | 63 (59–72) | 0.041 |
|  | H24 | 61 (52–64) | 1.000 |

*Data are presented as median (IQR).*

**FIGURES**


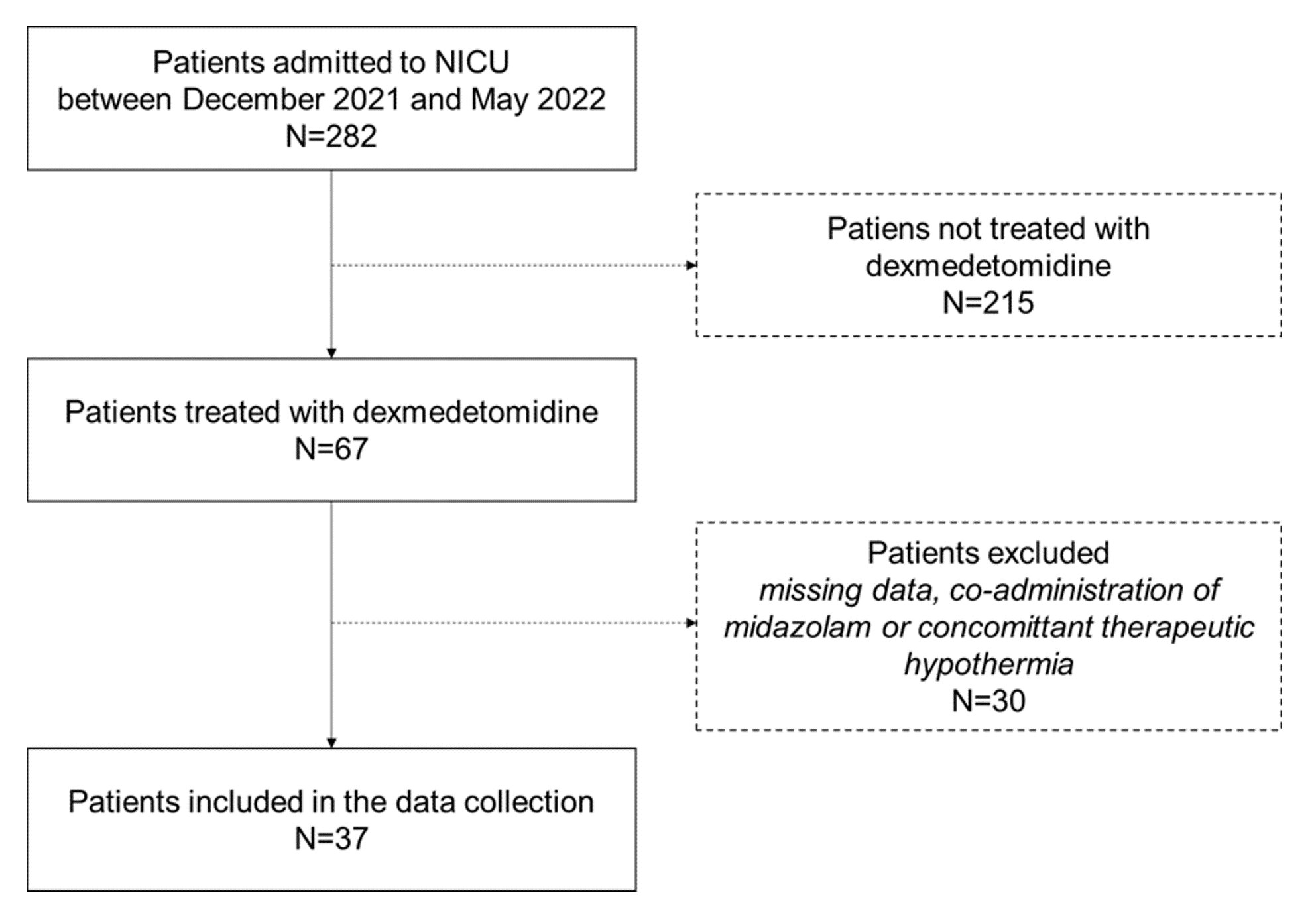


**Fig 1. Flow chart**


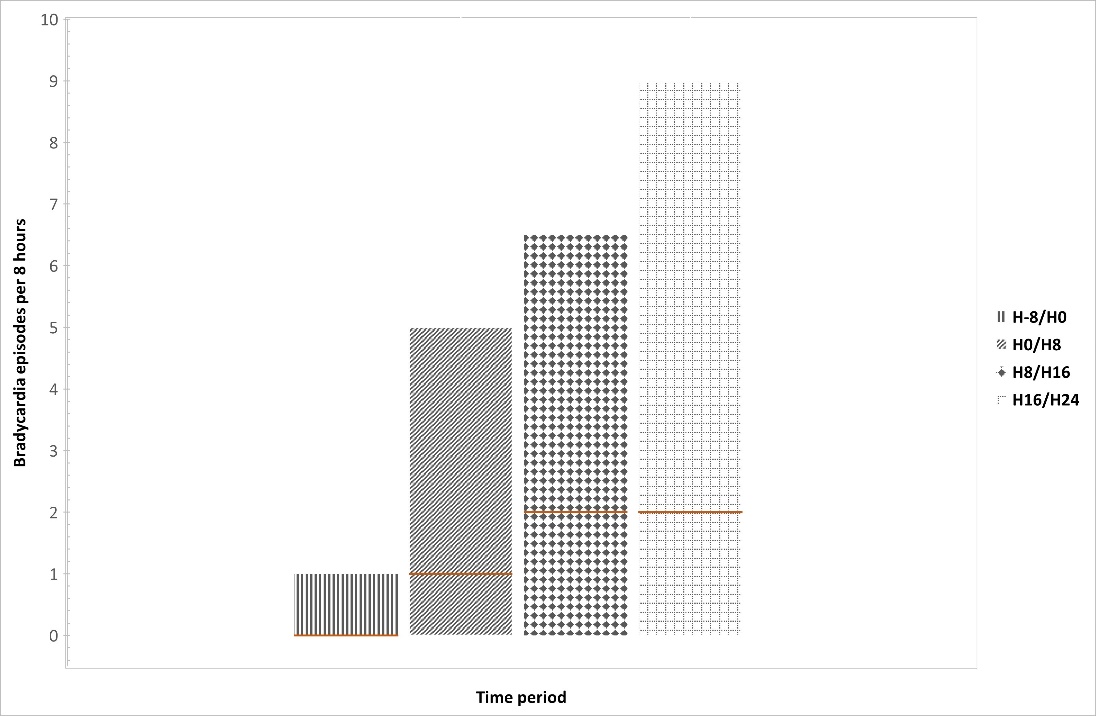


**Fig 2. Total bradycardia episodes before and after dexmedetomidine initiation**
Boxplots show the number of bradycardia episodes per 8-hour period during the 8 hours preceding treatment (H−8/H0) and the three consecutive 8-hour periods following initiation. Data are presented as median and interquartile range.

**Appendix 1. Analgesic sedation protocol with dexmedetomidine.**

Initiation of dexmedetomidine

- Start as a continuous infusion at 0.4µg/kg/h

Dosage adjustment of dexmedetomidine:

**1. According to comfort**

- If the EDIN score overtakes 5 if the Comfort Behavior score overtakes 18: increase the flow rate to 0.2 µg/kg/h steps, up to a maximum dose of 1.4 µg/kg/h.
- If the EDIN score falls below 4 or if the Comfort Behavior score falls below 11: first reduce morphine (or sufentanil) until stopped, then dexmedetomidine by 0.2 µg/kg/h.

**2. According to heart rate**

- Full-term newborn: reduce the flow rate by 0.2 µg/kg/h if the heart rate falls below 80 beats per minute.
- Premature neonate: reduce the flow rate by 0.2 µg/kg/h if the heart rate falls below 90 beats per minute.
